# Response boosts serial dependence in the numerosity estimation task

**DOI:** 10.1038/s41598-024-52470-0

**Published:** 2024-01-24

**Authors:** Yukihiro Morimoto, Shogo Makioka

**Affiliations:** 1 Department of Sustainable System Sciences, Osaka Prefecture University, 1-1, Gakuen-cho, Naka-ku, Sakai, Osaka 599-8531 Japan; 2 Department of Psychology, Osaka Metropolitan University, 1-1, Gakuen-cho, Naka-ku, Sakai, Osaka 599-8531 Japan

**Keywords:** Human behaviour, Decision, Visual system

## Abstract

Perceptions of current stimuli are sometimes biased toward or away from past perceptions. This phenomenon is called serial dependence. However, the strength of the effect of past responses on serial dependence has not been fully elucidated. We conducted experiments with a task in which participants estimated the number of dot arrays (numerosity estimation task) and directly compared whether the strength of serial dependence changed in the numerosity estimation task when participants responded or did not respond in the immediately preceding trial. We also examined whether the strength of serial dependence affected the accuracy of the numerosity estimation. We found that attractive serial dependence was stronger when participants responded in the immediately preceding trial than when they only saw the stimulus. The results suggest that the information from the previous stimulus must reach the higher-level processes associated with perceptual decisions to influence the estimation of the current stimulus. However, it is possible that the results of this study are specific to tasks in which participants respond with numeric symbols. The magnitude of the serial dependence effect was not observed to affect numerosity estimation performance, and no evidence was found that serial dependence enhances accuracy in the numerosity estimation task.

## Introduction

Previous perceptual experiences have a significant influence on current perceptions of the external world. One example of such an effect is the phenomenon known as serial dependence^1–5^, where the current perception of a stimulus is influenced by the previous perception of the stimulus. When the current perception is drawn towards the previous perception, it is referred to as “attractive serial dependence.” Conversely, when it is repelled, it is known as “repulsive serial dependence”^6,7^. Serial dependence shares similarities with the aftereffects that were studied before the discovery of serial dependence^8^. In this paper, unless otherwise specified, the term “serial dependence” refers to attractive serial dependence.

The study of serial dependence has been conducted across diverse tasks that employ varying types of stimuli, including orientation perception^2,7,9^, numerosity perception^1,10–13^, motion perception^14^, face perception^15^, sense of agency^16^, and monetary values^17^. Attractive serial dependence is believed to stabilize the perception of continuously changing stimuli in the presence of noise, such as blinking or occlusion by objects^9,18^. Repulsive serial dependence, also called adaptation, works to increase sensitivity to changes in the external environment and facilitates adaptation to new situations. These functions are considered to optimize our perception of the environment^6,19^. There have been various hypotheses proposed regarding the mechanism behind this phenomenon, including a continuity field that sustains visual stability and continuity^2^, feedback from high-level processing^20^, the effect of memory^21^ and response biases^7^, the influence of perceptual decision templates^6^, perceptual templates^22^, the active inference of vision^19,23^ and Bayesian inference^24^.

The question of whether serial dependence originates from low-level or high-level perceptual processing has been discussed. Findings in favor of low-level processing include the spatial localization of serial dependence ^2,12,25^, its occurrence in simple detection tasks ^26^, and it being influenced by the physical stimulus itself rather than perception, including illusions ^22^. Findings favoring high-level processing include the following: serial dependence requires attention^2,10^, is induced by different stimuli representing the same attribute^20,27^, requires conscious perception^28,29^, can be explained as a phenomenon of the decision-making process rather than an effect of past stimuli, and is affected by the proximity (similarity) in the feature space between the stimulus to respond and the preceding stimulus^7,30,31^. Moreover, confidence in task performance increases serial dependence^32^, and feedback on the task increases serial dependence^27^.

Considering that serial dependence has been confirmed for both low-level and high-level processing, the view that multiple processing stages contribute to serial dependence has recently become common. In particular, a study demonstrated that high-level prior information propagates to early sensory analysis^26^, suggesting that low-level and high-level processing do not independently contribute to serial dependence but rather influence each other. How perception and behavior are shaped by the interaction of prior and current information at multiple processing stages is now being discussed^3,5^.

The influence of serial dependence on numerosity has been examined primarily using an array of dots as a stimulus^1,10,12^. Participants perform a task to discriminate the numerosity of dots in the reference and probe stimuli^10,25^ or to estimate the numerosity of dots and input the number via keyboard^1,12^ The serial dependence in the numerosity discrimination task depends on the stimulus location, and the magnitude of the serial dependence effect does not differ between monocular and binocular conditions.^20,25,33^. These findings suggest that serial dependence in numerosity perception occurs in the region of the visual cortex where positional information is preserved, and binocular information is integrated. Furthermore, the attractive serial dependence becomes repulsive in experiments using backward masking, which interferes with feedback processing in the visual cortex^33^. It has also been reported that serial dependence in number discrimination tasks occurs even when there is no explicit task for the stimulus^25^. Serial dependence has also been reported to occur between different types of stimuli, such as the number of dots and number of screen flashes^20^ and the number of dots and digit number^27^. It has also been reported that feedback on task performance boosts serial dependence^27^. Serial dependence has also been confirmed for monetary values, which require high-level processing, such as calculation^17^. Taken together, these results suggest that both low-level visual processing (spatial localization) and high-level processing (abstract number) influence serial dependence effects in numerosity perception. Furthermore, as noted above, high-level and low-level processing are thought to interact.

There is also a finding that the response to a stimulus, rather than the past stimulus itself, influences serial dependence. In a task in which participants adjusted the orientation of a Gabor patch, the effect of serial dependence was attributed to the response to a past stimulus rather than to the orientation of the past stimulus^6^. When the orientation of a past stimulus affects the response to the current stimulus, the effect of the past response on the current response is also inevitably observed. This is because the response in a given trial is necessarily correlated with the orientation of the stimulus in that trial. However, if the past response has a stronger effect on serial dependence than the past stimulus, the response itself is considered to influence serial dependence. Indeed, it has been reported that the effect of past responses is greater than the effect of past stimuli in the serial dependence of orientation perception^6^ and monetary value^17^. Pascucci et al. noted that serial dependence is influenced not by the stimulus itself but by perceptual decisions about the stimulus and is related to decision inertia^6,34^. Notably, response involves not only perceptual decisions but also various processes, such as remembering the stimuli and motor output^35^. While it is difficult to specify exactly how the presence or absence of a response in a task alters the participant's internal processing, the involvement of higher-order processes is expected to be greater when a response is made than when no response is made.

It should be noted, however, that serial dependence is also observed after trials in which the subject did not make response^2^. In the Gabor patch orientation adjustment task, serial dependence is reported even immediately after trials in which the participant only saw the stimulus and was not required to respond^2,36^. In the numerosity discrimination task, many studies have confirmed that serial dependence is caused by a stimulus that does not require a response^10,25,27^. These findings suggest that attractive serial dependence cannot be explained by response history alone.

In tasks that require responses to stimulus quantities rather than a binary choice, such as adjusting the orientation of a Gabor patch or estimating monetary values, past responses have stronger effects than past stimuli. This suggested that whether serial dependence is strongly dependent on past stimuli or responses depends on the type of task. However, the relationship between the effect of response history and the nature of the task on serial dependence has not been elucidated.

Numerosity discrimination and numerosity estimation tasks are commonly used to investigate not only serial dependence but also numerosity perception itself^37,38^. The processing required for the two tasks differ from each other^39^. The discrimination task is considered to require a process in which one approximates and compares the numerosity of dots using the approximate number system (ANS)^38,40^. Processing by the ANS occurs at a relatively low level and is considered automatically driven by stimulus presentation in both tasks. In the numerosity estimation task, however, the approximated numerosity of dots needs to be converted into a specific number symbol and then entered via a keyboard^38^. The process of converting the approximated numerosity to number symbols occurs at a relatively high level and is only needed for numerosity estimation tasks. This difference is expected to affect whether the stimulus or the response history has a stronger influence on serial dependence.

**Figure 1:**
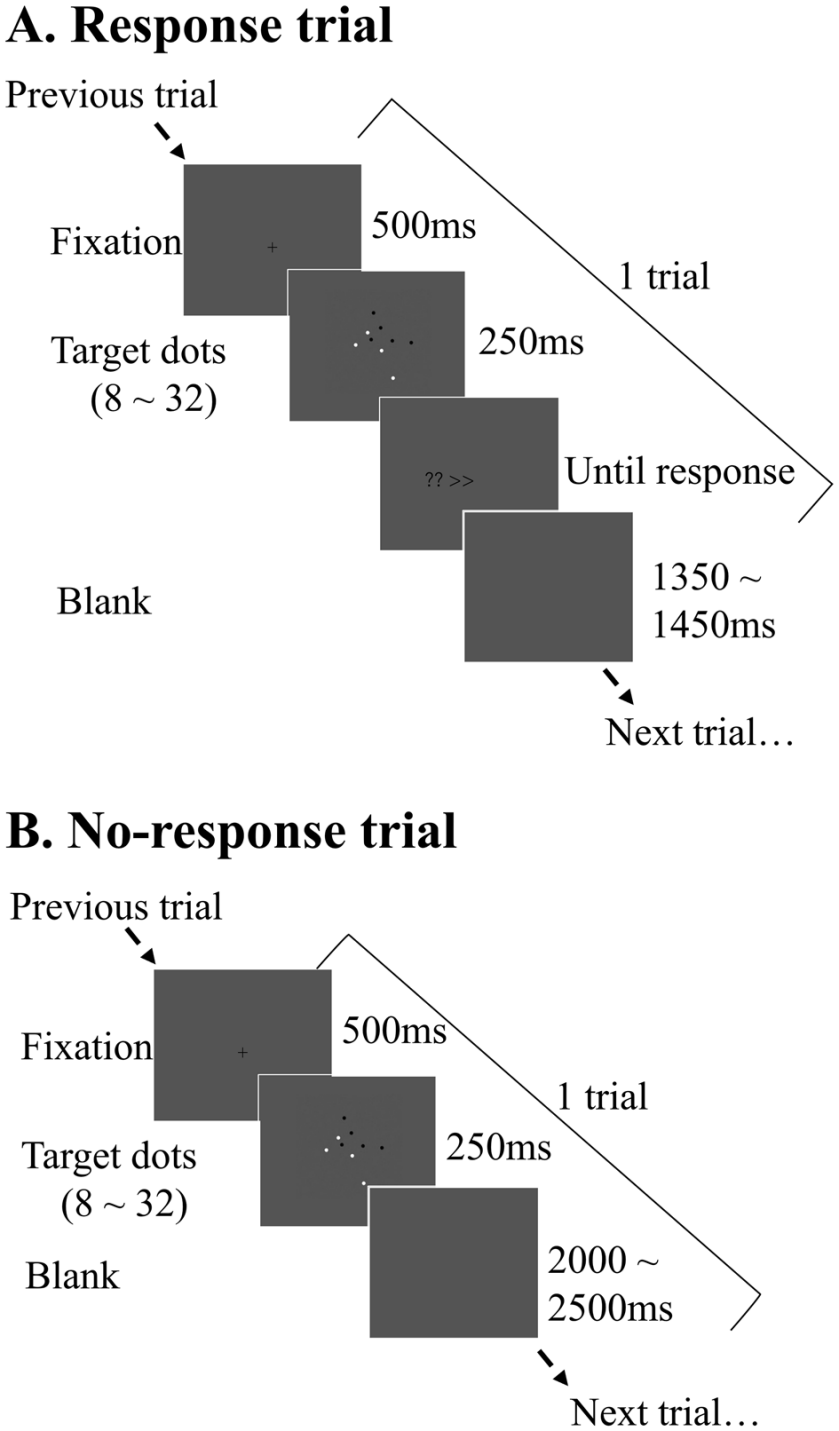
(**A**) Experimental procedure of the response trial. In each trial, a fixation cross was first presented for 500 ms, followed by the stimulus for 250 ms. The target stimuli included black and white dots, and the total number of dots ranged from 8 to 32. A response prompt was then presented. The participants were instructed to report the estimated number of dots by entering the number on a keyboard. Intertrial intervals varied randomly between 1350 and 1450 ms. (**B**) Experimental procedure of the no-response trial. The durations of the presentation of the fixation cross and stimulus were the same as those in the response trial. No response prompt was presented, and the intertrial intervals varied randomly between 2000 and 2500 ms. The participants simply viewed the stimulus, and no response was needed.

In this study, we used a numerosity estimation task and compared the strength of serial dependence immediately after the trial in which participants simply looked at an array with the strength of serial dependence immediately after a trial in which participants reported the number of dots (Fig. 1). If the strength of serial dependence does not change between these two conditions, it suggests that primarily low-level processing causes serial dependence in numerosity estimation since processing by the ANS occurs automatically by stimulus presentation. A stronger degree of serial dependence after the response suggests that serial dependence in numerosity estimation depends on high-level processing.

The primary aim of this study was to examine the effect of response history on the numerosity estimation task. We verified whether response history strengthens serial dependence by comparing serial dependence after trials in which participants simply viewed the stimulus (no-response trials) and after trials in which they responded to the stimulus (response trials). As noted earlier, the internal representations used for responses are considered to differ between the discrimination task and the numerosity estimation task. Comparing the results of this study with those in the discrimination task in the previous study provided a deeper understanding of the level of processing that causes serial dependence.

The second aim of this study was to examine whether serial dependence effects affect the accuracy of numerosity estimation. Although serial dependence is considered to keep our perceptions stable^2,3^, the extent to which serial dependence contributes to the accuracy of the estimation of the external environment has not been fully explored. In experimental settings where the order of stimulus presentation is random and not continuous, serial dependence would be disadvantageous in terms of accuracy. We examined whether the presence or absence of a response on the immediately preceding trial affects the accuracy of the estimation task.

### Statistical analysis

We assessed the general performance of Experiment 1A, which consisted entirely of response trials, and Experiment 1B, which alternated between no-response and response trials, by calculating the average of the participants’ estimates in each condition of the number of dots and the precision of the estimates in terms of Weber's fraction. We also examined serial dependence using the same procedure as employed by previous study^12^. The strength of serial dependence was determined by the degree to which the response in the current trial (n) was influenced by the number of dots in the immediately preceding trial (n − 1) or by the number of dots in earlier trials (n − 2, n − 3, up to n − 6). The estimation error (response—true number of dots) was first calculated, and a linear regression was performed for each participant with the estimation error as the objective variable and the number of dots in the immediately preceding trial as the explanatory variable. Positive errors indicated overestimation of the number of dots in the current trial, while negative errors indicated underestimation of the number of dots in the current trial. The slope of the linear fit represents the strength of the attractive serial dependence effect. A positive slope means that as the number of dots in the immediately preceding trial increases, the estimation error in the current trial increases in the positive direction (attractive serial dependence). Conversely, a negative slope indicates a repulsive effect (repulsive serial dependence). In Experiment 2, the order of no-response and response trials was randomized. The analysis was the same as in Experiment 1b.

We conducted one-sample *t* tests against the null hypothesis that the mean value of the slopes did not differ from zero to evaluate the significance of the serial dependence effects in both Experiments 1 and 2. To control the probability of type I errors in multiple comparisons, the false discovery rate (FDR) was calculated, and the FDR-corrected significance level was 5% in all analyses.

## Results

### Experiment 1

In Experiment 1, we examined whether responses are required for serial dependence effects in the numerosity estimation task by comparing serial dependence after trials in which participants simply viewed the stimuli and after trials in which they responded to the stimuli. Experiment 1a consisted of 704 trials, in which participants estimated the numerosity of dots in the array and responded in all trials. Experiment 1b also consisted of 704 trials, and in half of the trials, participants were not required to respond but only to see the dot array (no-response trials). In the other half of the trials, participants responded by estimating the numerosity of dots in the array (response trials). The no-response and response trials were alternated. The same participants participated in Experiments 1a and 1b (*N* = 33). The order of Experiments 1a and 1b was counterbalanced across participants.

**Figure 2:**
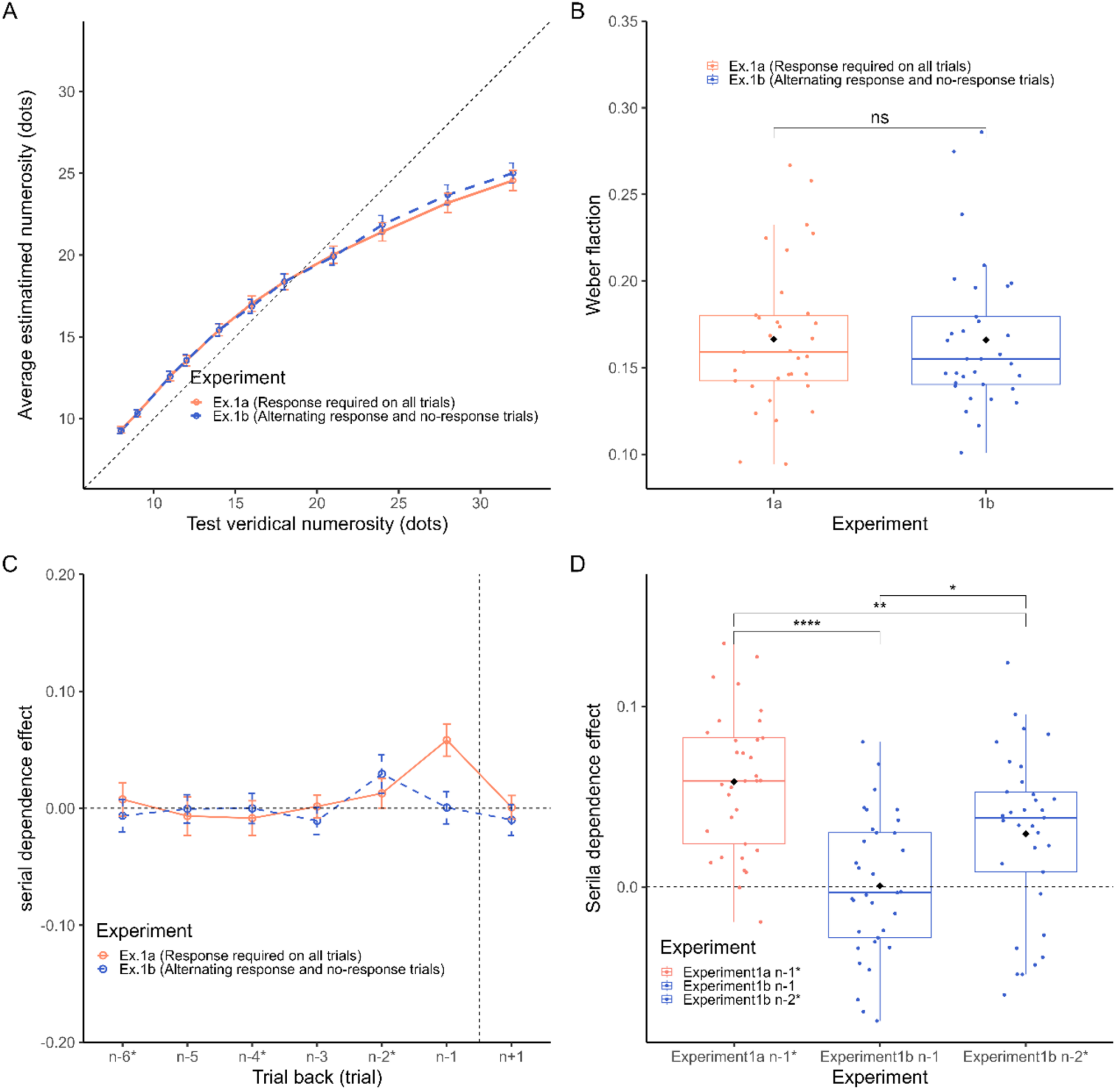
(**A**) Average numerical estimates at each level of the numerosity range of Experiments 1a and 1b. (**B**) Average Weber’s fraction across the two experiments. (**C**) Serial dependence effects in Experiments 1a and 1b. Serial dependence effects induced by the immediately previous stimulus (n − 1) and stimuli further back in the past (n − 2, n − 3, to n − 6), with the future trial (n + 1) as a control. (**D**) Comparison of serial dependence effects between Experiments 1a and 1b. In Experiment 1b, response and no-response trials were alternated. When a response was made in the current trial, no response was made in the n − 1 trial, and a response was made in the n − 2 trial. Therefore, we compared the serial dependence effects of the number of dots in the n − 1 and n − 2 trials in Experiment 1b. Error bars in (**A**) are SEM, (**B**, **C**, **D**) is 95% CI. Significace levels in the figures are FDR-adjusted *p* values; ns = not significant, *p < 0.05,**p < 0.01, ***p < 0.001.

First, we assessed participants’ general performance in the numerosity estimation task. Figure 2A shows the average estimated number of dots at each level of numerosity (8–32). The distribution of errors per participant is shown in the Supplementary Information. The change in the average estimated number of dots with veridical numerosity was similar between Experiments 1a and 1b. A two-way repeated-measures ANOVA on estimated numerosity with factors “numerosity” (i.e., the different levels of the numerosity) and “experiment” (i.e., Experiment 1a in which participants responded all trials vs. Experiment 1b in which response trials were alternated with no-response trials) showed only the main effect of “numerosity” (*F* (1.34,42.80) = 661.072, *p* < 0.001, $\eta^{2}_{p}$ = 0.954), with no effect of “experiment” (*F* (1,32) = 0.168, *p* = 0.685) or interaction (*F* (3.56,113.84) = 1.86, *p* = 0.13). We then assessed the average Weber’s fraction across the different levels of numerosity following the procedure performed by Fornaciai and Park^27^, computed as the standard deviation of answer estimation at each level divided by the average answer estimation (Fig. 2B). No significant difference was observed in the average Weber’s fractions (paired *t* test, *t*(32) = 0.088, *p* = 0.9305, *d* = 0.01). This means that there was no significant difference in precision between Experiments 1a and 1b.

Figure 2C shows the serial dependence effects due to the number of dots on the preceding and following trials. Serial dependence was assessed individually for each participant and condition by regression analysis with the number of dots in the stimulus array as the explanatory variable and the estimation error as the objective variable. Significant positive average effects indicated attractive serial dependence, while significant negative average effects indicated repulsive serial dependence.

In Experiment 1a, in which participants responded on all trials, the most prominent attractive serial dependence effect was provided by the previous (n − 1) trial stimulus, while the effect of stimuli further in the past approached zero. We performed a series of one-sample *t* tests against zero, corrected for multiple comparisons using the false discovery rate (FDR)^41^ procedure (*q* = 0.05), and observed a significant serial dependence effect at n − 1 (*t*(32) = 8.65, adjusted-*p* < 0.001, *d* = 1.51). No other effect reached significance after FDR correction (all adj-*p* > 0.05). See Supplementary Information for details of the analyses.

In Experiment 1b, in which response trials were alternated with no-response trials, participants always only saw the stimulus but did not respond in n − 1 trials. On the other hand, in the n − 2 trial, participants always responded. We only observed a statistically significant effect at n − 2 (*t*(32) = 0.029, adj-*p* < 0.001, *d* = 0.638), and no other significant influence from stimuli further in the past was observed (all adj-*p* > 0.05). To assess the difference between Experiment 1a and 1b at n − 1, we performed a paired *t* test, which showed a statistically significant difference (*t*(32) = 5.92, *p* < 0.001,* d* = 1.48). The effect of attractive serial dependence was significantly greater when the response was made on the immediately preceding trial.

Finally, we also assessed the influence provided by the stimulus in the future trial (n + 1) and current trial (n) on the current error. The effect at n + 1 was not significantly higher than zero in either Experiment 1a or 1b (Experiment 1a: *t*(32) = 0.155, adj-*p* = 0.877, *d* = 0.02; Experiment 1b: *t*(32) = 0.248, adj-*p* = 0.989, d = 0.04). The effect at n was significantly higher than zero in both Experiment 1a and 1b (Experiment 1a:* t*(32) = − 13.242, adj-*p* < 0.001, *d* = − 2.305; Experiment 1b: *t*(32) = − 12.93, adj-*p* < 0.001, *d* = − 2.252). The negative effect of the number of dots in the current trial reflected the tendency for underestimation to be greater as the number of dots in the stimulus array increased (Fig. 2A). This effect is usually seen in tasks in which participants estimate the number of stimuli^12,27^.

The lack of serial dependence by the number of dots in the n − 1 trials in which no response was made and the significant serial dependence by the number of dots in the n − 2 trials in which a response was made in Experiment 1b suggested that for serial dependence to occur in the task of estimating the number of dots, the stimulus must be responded to, not just seen.

### Experiment 2

In Experiment 1b, no-response and response trials always alternated; thus, it was possible that participants intentionally or unintentionally ignored the stimuli in the no-response trials because they did not have to respond in that trial. To address this issue, the order of no-response and response trials was randomized in Experiment 2. Participants (*N* = 23) did not know whether they needed to respond until the response prompt was presented; thus, they had to attend to the stimulus. Experiment 2 was composed of 2 blocks of 176 response trials and 2 blocks of 176 no-response trials.

**Figure 3:**
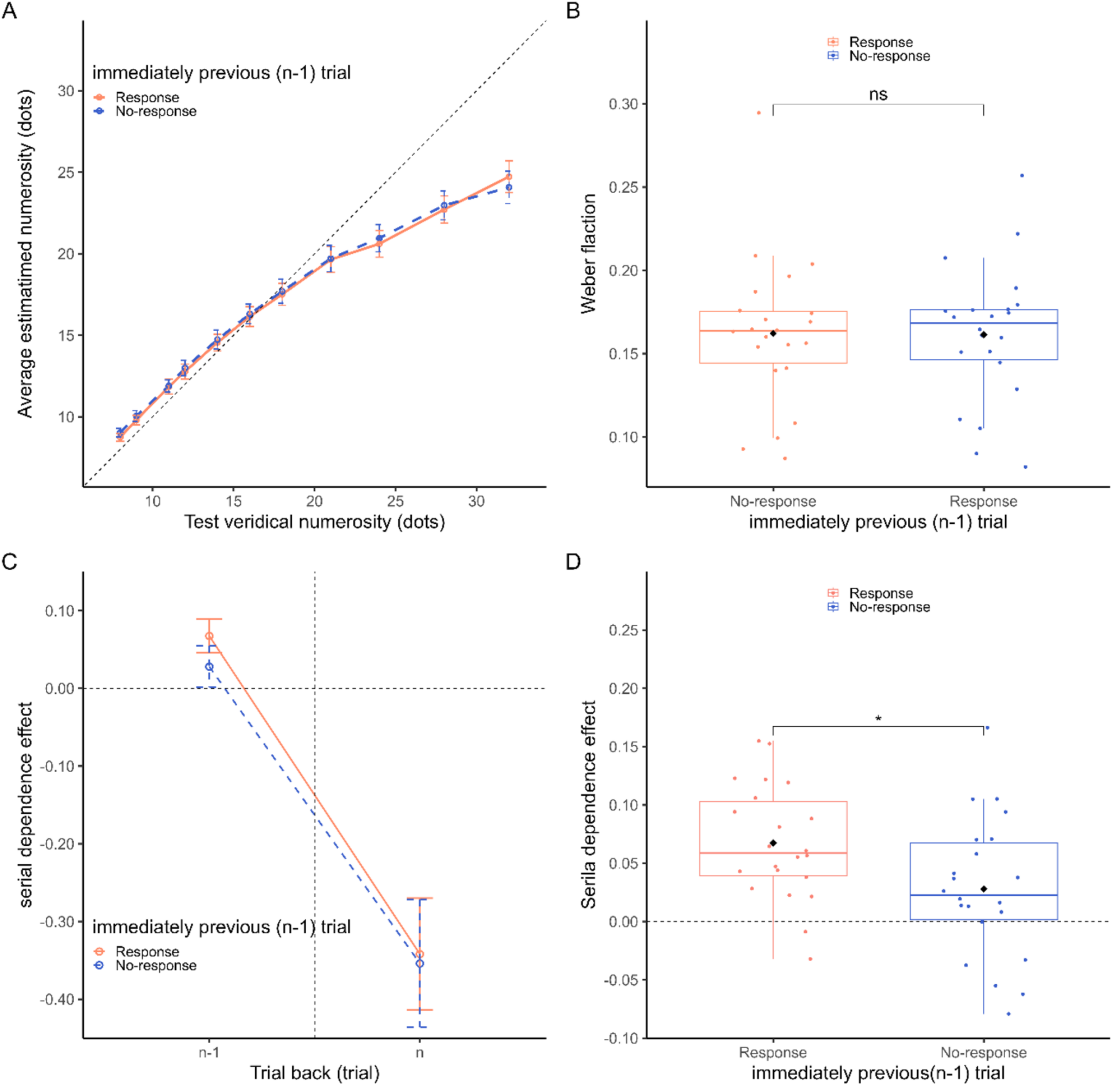
(**A**) Average numerical estimates at each level of the numerosity range of Experiment 2. (**B**) Average Weber’s fraction (WF) classified by whether the immediately previous trial required a response. (**C**) Serial dependence effects classified by whether the immediately previous trial required a response. The graph shows the serial dependence effect of the number of dots in the immediately preceding (n − 1) trial and the effect of the number of dots in the current trial as a control condition. (**D**) Distribution of serial dependence effects for each participant, classified by whether the immediately previous trial required a response. Error bars in (**A**) are the SEM, those in (**B**, **C**, **D**) are the 95% CIs. Significance levels in the figures are FDR-adjusted *p* values; ns = not singnificant, *p < 0.05, **p < 0.01, ***p < 0.001.

Figure 3A shows the average estimated number of dots at each level of numerosity (8–32). The change in the average estimated number of dots with veridical numerosity was similar regardless of whether the immediately preceding trial required a response. A two-way repeated-measures ANOVA on estimated numerosity with factors “numerosity” (i.e., the different levels of the numerosity) and “condition” (i.e., whether the immediately preceding trial required a response) showed only the main effect of numerosity (*F* (1.64,34.20) = 269.111, *p* < 0.001, $\eta^{2}_{p}$ = 0.928), with no effect of “condition” (*F* (1,21) = 1.324, *p* = 0.263) or interaction (*F* (5.52,115.91) = 0.966, *p* = 0.44). We then assessed the average Weber’s fractions (Fig. 3B) across the conditions. No significant difference was observed in the average Weber’s fractions (paired *t* test, *t*(21) = 0.191, *p* = 0.850, *d* = 0.02). This means that there was no significant difference in precision between the conditions.

The effect of serial dependence was derived in the same way as in Experiments 1a and 1b. The average serial dependence effects induced by the previous trial stimulus (n − 1) are shown in Fig. 3C. When the immediate previous trial demanded a response, we observed a significant serial dependence effect at n − 1 (*t*(21) = 2.25, adjusted-*p* < 0.001, *d* = 1.37). When no response was requested on the immediate previous trial, a significant serial dependence effect at n − 1 was not observed (*t*(21) = 2.17, adjusted-*p* = 0.08, *d* = 0.463). To assess the difference between when a response was requested on the immediately preceding trial and when it was not, we performed a paired *t* test, which revealed a significant difference (*t*(21) = 5.92, *p* = 0.04, *d* = 0.716).

Finally, we assessed the influence provided by the stimulus in the current trial (n). The effect was significantly higher than zero (*t*(21) = − 9.89, adj-*p* < 0.001, *d* = − 2.108 and *t*(21) = − 8.98, adj-*p* < 0.001, *d* = − 1.914, respectively). This may reflect an increasing tendency toward underestimation as the number of dots increases.

As in Experiment 1, the effect of serial dependence due to the number of dots in the immediately preceding trial was significant when a response was requested and not significant when a response was not requested. However, the probability of type I error when no response was requested was below 10% (adjusted *p* = 0.08), making it difficult to claim that serial dependence did not occur at all. When comparing the two conditions, as in Experiment 1, the effect of serial dependence was significantly stronger when a response was needed.

## Discussion

Attractive serial dependence has been reported to occur with a variety of stimuli, including line orientation^2^, motion^14^, faces^42^, numerosity perception^1,10^, and sense of agency^16^. These phenomena are similar in that they make the experience of the current stimulus similar to that of the past. However, the representations that cause serial dependence are thought to vary from stimulus to stimulus. In the Gabor patch orientation adjustment task, information held in the perceptual system is thought to influence the orientation judgment of the current stimulus^2,26^. In the estimation of the numerosity of dots, the numerosity is supposed to be estimated by a system specific to numerosity perception^43^, using the density and number of stimuli and the area the dots occupy in the visual field as cues. On the other hand, the task of estimating the numerosity of dots and responding with a number requires more abstract and higher-level processing than the comparison of the numerosity of dots in the stimuli^38,39^. The fact that serial dependence is observed in a variety of tasks, despite the different levels of processing, suggested that this phenomenon reflects a universal property of the system to estimate features of the external world. The primary aim of this study was to examine the effect of response history on the numerosity estimation task. We verified whether response history strengthens serial dependence by comparing serial dependence after trials in which participants simply viewed the stimulus and after trials in which they responded to the stimulus. In the response trials, the numerosity of dots in the stimulus had to be estimated and converted into an abstract representation of a number (number symbol) before the response, whereas in the no-response trial, the number did not have to be converted into a number symbol. We examined whether the effect of serial dependence differed depending on the processing required in the preceding trial.

In Experiment 1a, in which a response was required in all trials, the effect of attractive serial dependence was observed, as was the case in the previous study. In Experiment 1b, on the other hand, there was no serial dependence effect due to the number of dots in the immediately previous (n − 1) trial. Furthermore, the mean effect of serial dependence was larger when the response was made on the immediately preceding trial (Experiment 1a) than when it was not (Experiment 1b). This implies that estimating the numerosity of dots and converting it to a number symbol strengthens the effect of serial dependence. An attractive serial dependence effect was observed based on the number of dots in n − 2 trials in Experiment 1b. This should be because the response was always absent in the n − 1 trial and present in the n − 2 trial.

These results were generally replicated in Experiment 2, with stronger serial dependence when responses were made in the immediately preceding trial than when they were not. These results are consistent with previous research showing that the magnitude of the response causes stronger serial dependence than the magnitude of the previous stimulus. The findings that serial dependence is strengthened by feedback^27^ and that it is strengthened when confidence is high^32^ are also consistent with the results of the present study in that internally generated representations, rather than stimulus input itself, influence the following estimation.

No significant serial dependence effect was observed immediately after the no-response trial in either Experiment 1b or Experiment 2; however, the significance level in Experiment 2 was less than 10%; thus, we cannot conclude that no effect exists after the no-response trial (see Figs. 2D and Fig. 3D). The difference in the results between Experiment 1b and Experiment 2 can be attributed to the different order of the trials in Experiments 1b and 2. In Experiment 1b, the no-response and response trials alternated, but in Experiment 2, the order of the trials was randomized so that participants did not expect whether they were to respond when the dot array was presented. We conjecture that in Experiment 2, some participants may have estimated the numerosity of dots when they saw the dot array and replaced them with numerical symbols, regardless of whether they were required to respond. This may have caused some degree of serial dependence effect.

In a previous study using the numerosity discrimination task, a significant serial dependence effect was noted in trials in which participants only saw the stimulus but did not respond^10^. In the numerosity discrimination task, participants saw the induction, reference, and probe stimuli and answered whether the reference or probe stimulus had more dots^10^. In this task, participants needed only to compare the numerosity of dots and did not need to convert the numerosity into a symbol. The results of previous studies suggest that the mere visual observation of a stimulus activates numerosity representations and causes serial dependence. On the other hand, the results of Experiments 1b and 2 suggest that activation of numerosity representations alone does not cause serial dependence in numerosity estimation tasks in which participants had to respond using number symbols. These results suggest that the information from the previous stimulus must reach the higher-level processes associated with decision making to influence the estimation for the current stimulus. The higher-level processes can include various stages, such as approximate numerical representation in the ANS or symbolic number representation. It is difficult to determine from the results of this experiment which stages contribute to the occurrence of serial dependence in numerosity estimation.

Another possible explanation for the difference in serial dependence effects on the no-response trials between the numerical discrimination task and the numerical estimation task could be the difference in the procedures. In the numerical estimation task in this study, immediately after the dot array was presented, a prompt was presented on the response trials but no prompt was presented on the no-response trials. Therefore, participants were able to know immediately after seeing the dot array whether a response was needed and thus did not need to retain the information about the stimulus. Therefore, the stimuli in the no-response trials could not have been fully processed. Further investigation is needed to determine whether the differences in the effects of the presence or absence of responses in the numerical discrimination and numerical estimation tasks are due to differences in the stage of processing to the stimuli or to differences in the procedures.

The second aim of this study was to examine whether serial dependence effects influence the accuracy of numerosity estimation. In both Experiments 1 and 2, we found no evidence that the strength of serial dependence affects the accuracy of numerosity estimation (see Figs. 2A and 3A). These results suggest that serial dependence plays neither a beneficial nor a detrimental role in the accuracy of the numerosity estimation task. This result is consistent with the results of a previous study in which the effect of serial dependence increased due to feedback on participants’ responses, but the effect on the accuracy of numerosity estimation was not observed^27^.

It is known that when the number of dots is small, participants can answer accurately by subitizing, but as the number increases, the error increases linearly. Serial dependence makes the estimate of the number of dots in the current stimulus closer to the past stimulus or response. When the number of objects in the environment varies continuously, serial dependence is expected to enable estimation of the number of objects at a lower cost. This is a similar idea to the decision template for orientation perception of Pascucci et al.^6^, which is consistent with the idea that serial dependence contributes to the stability of the visual world and adaptation to a continuously changing environment^3,4^. However, in the experiments used to study serial dependence, the number of dots does not vary continuously but varies randomly within a certain range. In this case, the strength of serial dependence would not affect the accuracy of numerosity estimation, since the number of dots in the previous trial does not provide a cue to improve the accuracy of the current trial's estimation. Future research on the potential function of serial dependence will require rigorous modeling of the changes occurring in the environment, followed by experiments to examine when serial dependence has advantages or disadvantages in the model environment.

## Conclusion

We examined whether the effect of serial dependence differed after trials in which participants responded and after trials in which they only looked at the stimulus in the numerosity estimation task. We found attractive serial dependence after trials in which participants responded immediately before but no significant serial dependence after trials in which participants only looked at the stimulus immediately before. These results suggest that the information from the previous stimulus must reach the higher-level processes associated with perceptual decisions to influence the estimation of the current stimulus. However, it is possible that the results of this study are specific to the task in which participants respond with numeric symbols.

The magnitude of the serial dependence effect was not observed to affect number estimation performance, and no evidence was found that serial dependence enhances accuracy in the numerosity estimation task. Future studies should examine the differences in the occurrence of serial dependence effects among tasks within numerosity perception and the nature of serial dependence in more detail by creating an experimental environment that contains variations more similar to the natural environment.

## Methods

We investigated serial dependence in two experiments using dot arrays as stimuli. In Experiment 1a, participants reported the estimated number of dots presented on the screen in all trials. In Experiment 1b, a trial in which participants simply saw the dots on the screen (no-response trial) alternated with a trial in which they reported the estimated number of dots (response trial). The same participants participated in both Experiments 1a and 1b successively on the same day. The order of the experiments was counterbalanced across participants. In Experiment 2, no-response trials and response trials were conducted in random order.

### Experiment 1a

#### Participants

A total of 35 subjects participated in the study (8 males, mean age = 19.19, SD = 1.33 years). The number of participants was determined based on the number of participants in similar studies^27^. Two of the participants were excluded from the analysis because they demonstrated insufficient understanding of the experimental task. Participants were compensated with a ¥2,000 fee or course credits for their participation. All participants had normal or corrected-to-normal vision and provided written informed consent prior to taking part in the study. All the experimental procedures were approved by the Ethics Committee of the Graduate School of Sustainable System Sciences, Osaka Metropolitan University and were performed in accordance with the latest version of the Declaration of Helsinki.

#### Apparatus and stimulus

All experiments were performed using MATLAB (version 2021a, The MathWorks Inc., Natick, MA, USA) and Psychtoolbox-3^44–46^ running on a computer with an Intel Core 7–8700 CPU and a GeForce GTX 1080Ti 11 GB GDDR5X GPU with an Ubuntu 20.04LTS operating system. Participants were presented with stimuli at a viewing distance of approximately 50 cm from the 24-inch monitor (ASUS VG248QE; resolution: 1920 × 1080 pixels). The refresh rate of the monitor was set to 99.89 Hz, and a USB keyboard was used to acquire responses.

The screen background was gray, and the instructions were presented in white font. The fixation cross (+) was black (20 × 20 pixels). All stimuli were arrays of black and white dots presented on a gray background. Each dot was randomly positioned within a virtual circular area, with possible positions only constrained by keeping a minimum interdot distance equal to at least the radius of one dot. The stimulus parameters were set as follows. Dot-array stimuli included 8, 9, 11, 12, 14, 16, 18, 21, 24, 28, or 32 dots. Regarding the other nonnumerical dimensions, the minimum individual dot diameter was 0.64 deg (20 pixels), while the maximum individual dot diameter was 1.27 deg (40 pixels); diameter varied with four steps of 0.22 deg (7 pixels) each. The minimum diameter of the virtual circular area where the dots were drawn was 11.74 deg (370 pixels), while the maximum diameter was 23.23 deg (740 pixels); diameter varied with four steps of 3.87 deg (123 pixels) each. In all cases, the individual dot size was kept equal within an array.

#### Procedure

The experimental procedure was designed with reference to that reported by Fornaciai and Park^12^. Participants performed a numerosity estimation task (Fig. 1A). In each trial, a fixation cross was first presented in the center of the screen for 500 ms, followed by a target array consisting of multiple dots for 250 ms. Then, the prompt “? >  > ” was presented. The participants were instructed to report the estimated number of dots by entering the number on a keyboard. No time limit was imposed on the response period. The numbers entered by the participants were shown on the screen, and they could correct their responses by pressing the backspace key. Once the input was complete, participants were instructed to press the enter key to confirm the response, and the next trial started automatically. The duration that the blank screen was displayed varied randomly in the range of 1350–1450 ms. Participants were informed of the range of the number of dots in the array in advance. To provide uncertainty at both ends of the range, a wider range (between 6 and 40) than the actual number of dots (between 8 and 32) was given.

Participants completed 4 blocks of 176 trials, for a total of 702 trials. Stimulus images were generated for each experiment, and the same image was never presented more than once. For each participant, stimuli consisting of one of the possible numbers of dots 8–32 (11 sets of numbers) were presented 16 times in each block. Participants were allowed to take a break of at least 30 s for each of the 88 trials. To exclude typing errors from the analysis, responses less than 0 or greater than 50 were excluded from the analysis.

Before starting the experimental trials, participants completed 25 practice trials. The procedure of the practice trials was identical to that of the experimental trials. Participants completed Experiments 1a and 1b on the same day; the total duration of the two experiments was approximately 120 min.

### Experiment 1b

#### Participants, apparatus, and stimuli

The participants, dot images, and apparatus were the same as in Experiment 1a.

#### Procedure

The procedure of Experiment 1b was the same as that of Experiment 1a, except that the trials in which participants only saw the stimulus and did not respond (no-response trial) alternated with trials in which participants reported the number of dots they estimated (response trial). The procedure of the response trials was the same as that of Experiment 1a. In the no-response trial, the stimulus was presented for 250 ms, and then a blank screen was presented for 2400–2500 ms before proceeding to the next trial.

We assigned half of the 704 stimuli used in Experiment 1 to the no-response trials and the rest to the response trials. One block consisted of 176 no-response trials and 176 response trials. Each block always began with a no-response trial, alternated between no-response and response trials, and ended with a response trial. A break was given every 88 trials.

### Experiment 2

#### Participants

A total of 23 subjects participated in the study (8 males, mean age = 18.65, SD = 1.13 years). Based on the comparison of serial dependence effects in Experiments 1a and b, the sample size was calculated by G*power^47,48^ with an effect size of 1.5, a significance level of 5%, and a power of 99%, which yielded a value of 11. The sample size was doubled because it was necessary to test twice, once after a trial with a response and once after a trial without a response, to verify whether the serial dependence effect significantly differed from 0. One participant was excluded from the analysis because they demonstrated insufficient understanding of the experimental task based on their data. Participants were compensated with a ¥1500 fee for their participation. All participants had normal or corrected-to-normal vision and provided written informed consent prior to taking part in the study. All the experimental procedures were approved by the Ethics Committee of the Graduate School of Sustainable System Sciences, Osaka Prefecture University and were performed in accordance with the latest version of the Declaration of Helsinki.

#### Apparatus and stimulus

The stimuli and apparatus were the same as in Experiment 1a.

#### Procedure

The experimental procedure was identical to Experiment 1b, except that the order of the no-response and response trials was randomized. The total duration of the experiment was approximately 90 min.

### Supplementary Information

Supplementary Information.

## Data Availability

The datasets collected in this study are available from the corresponding author on reasonable request.
