# Supplementary material for: Response boosts serial dependence in the numerosity estimation task
Source: Sci Rep. 2024 Jan 24;14:2059. doi: 10.1038/s41598-024-52470-0 (PMC10808238; doi:10.1038/s41598-024-52470-0)
Supplement: Supplementary file 1 — Supplementary Information. [file 41598_2024_52470_MOESM1_ESM.docx]

**Supplementary Information**


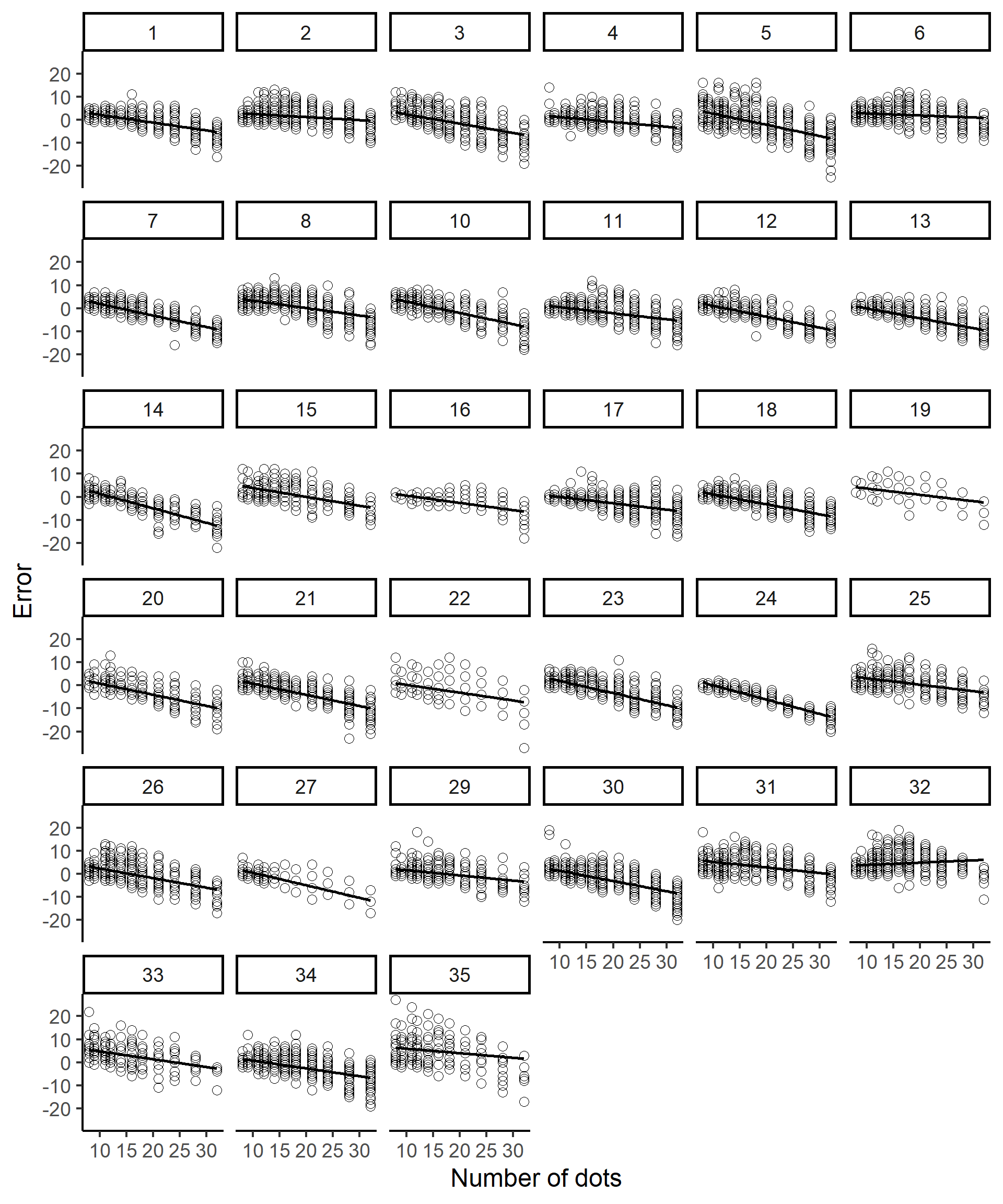


# **Figure S1.** Distribution of errors as a function of the number of dots in the current trial in Experiment 1a (all trials required a response). The circles in the graph indicate the error value for each participant on each trial. The numbers in the rectangles represent the participant number. The lines in the graphs indicate the slopes obtained from regression analyses.


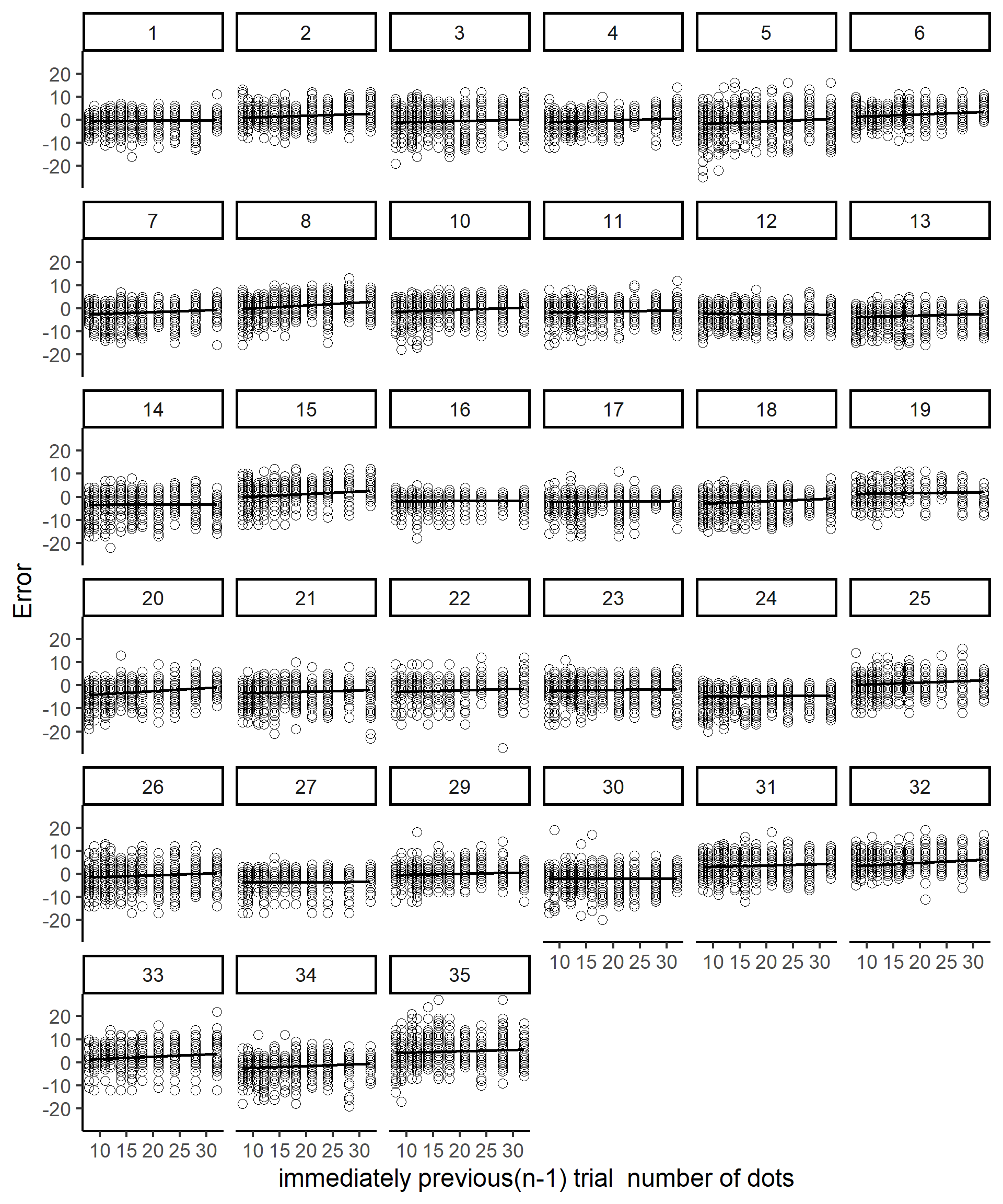


# **Figure S2.** Distribution of errors as a function of the number of dots in the n-1 trial in Experiment 1a (all trials required a response). The circles in the graph indicate the error value for each participant on each trial. The numbers in the rectangles represent the participant number. The lines in the graphs indicate the slopes obtained from regression analyses.


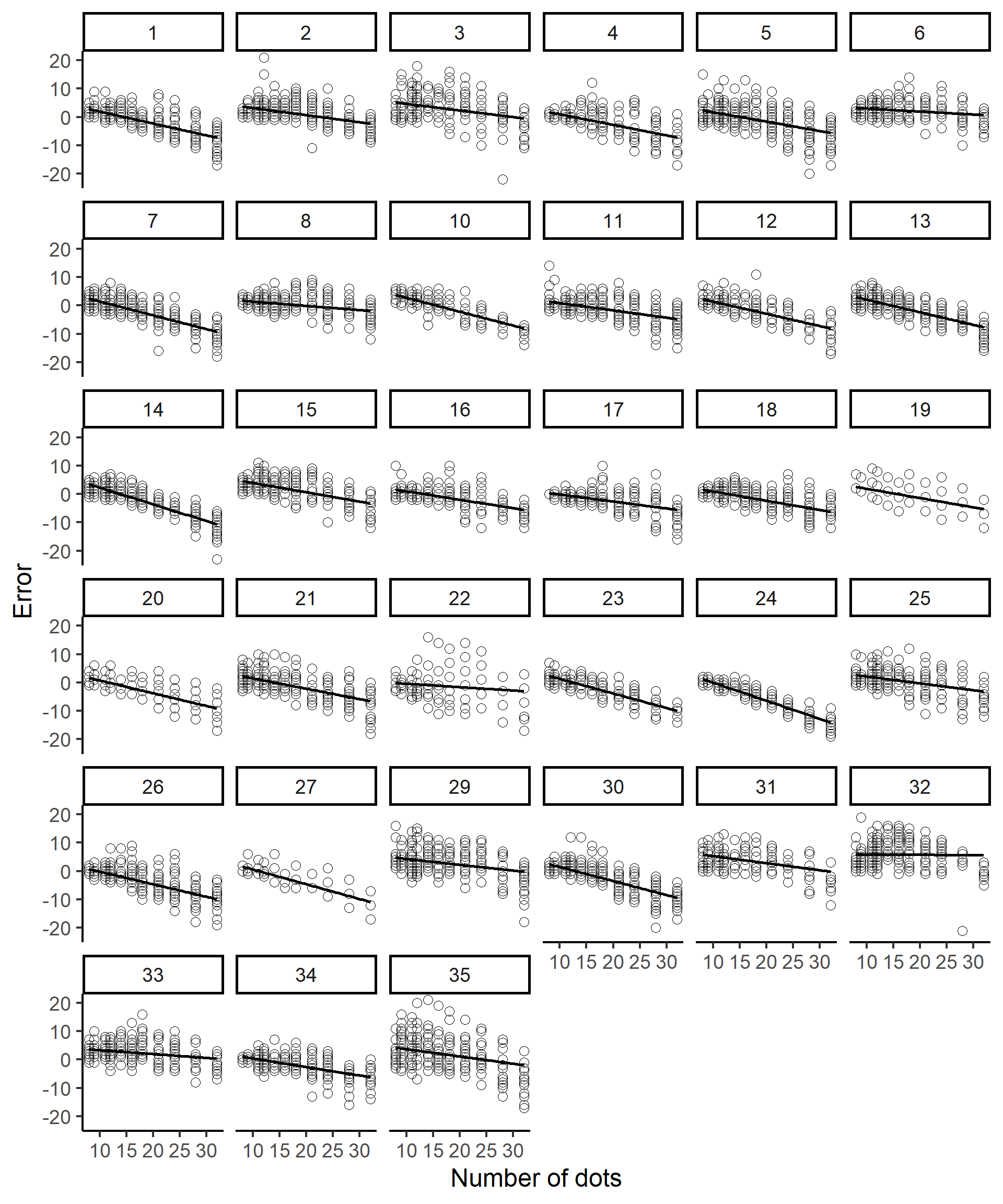


# **Figure S3.** Distribution of errors as a function of the number of dots in the current trial in Experiment 1b (response and no-response trials were alternated). The circles in the graph indicate the error value for each participant on each trial. The numbers in the rectangles represent the participant number. The lines in the graphs indicate the slopes obtained from regression analyses.


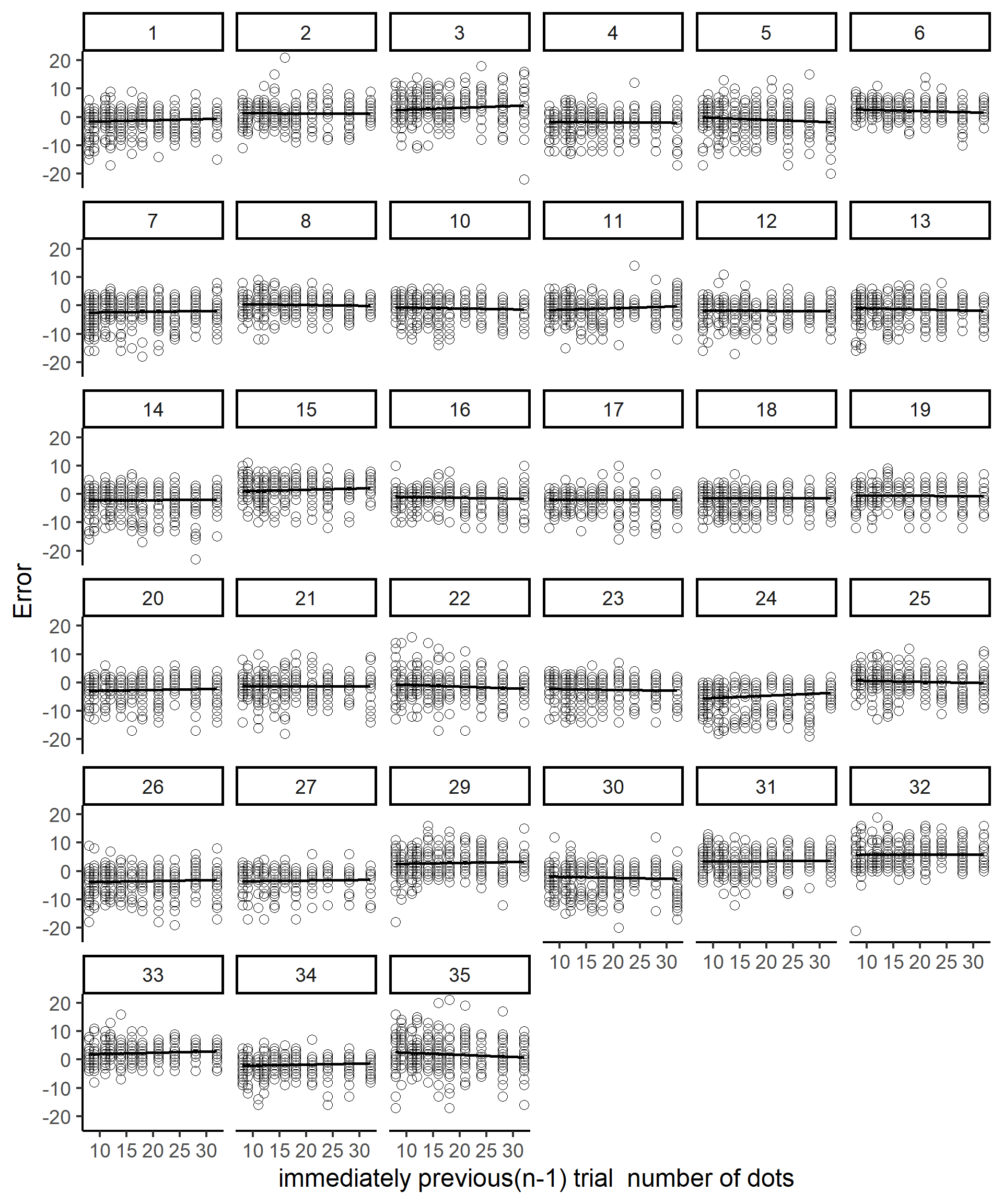


# **Figure S4.** Distribution of errors as a function of the number of dots in the n-1 trial in Experiment 1 b (response and no-response trials were alternated). The circles in the graph indicate the error value for each participant on each trial. The numbers in the rectangles represent the participant number. The lines in the graphs indicate the slopes obtained from regression analyses.


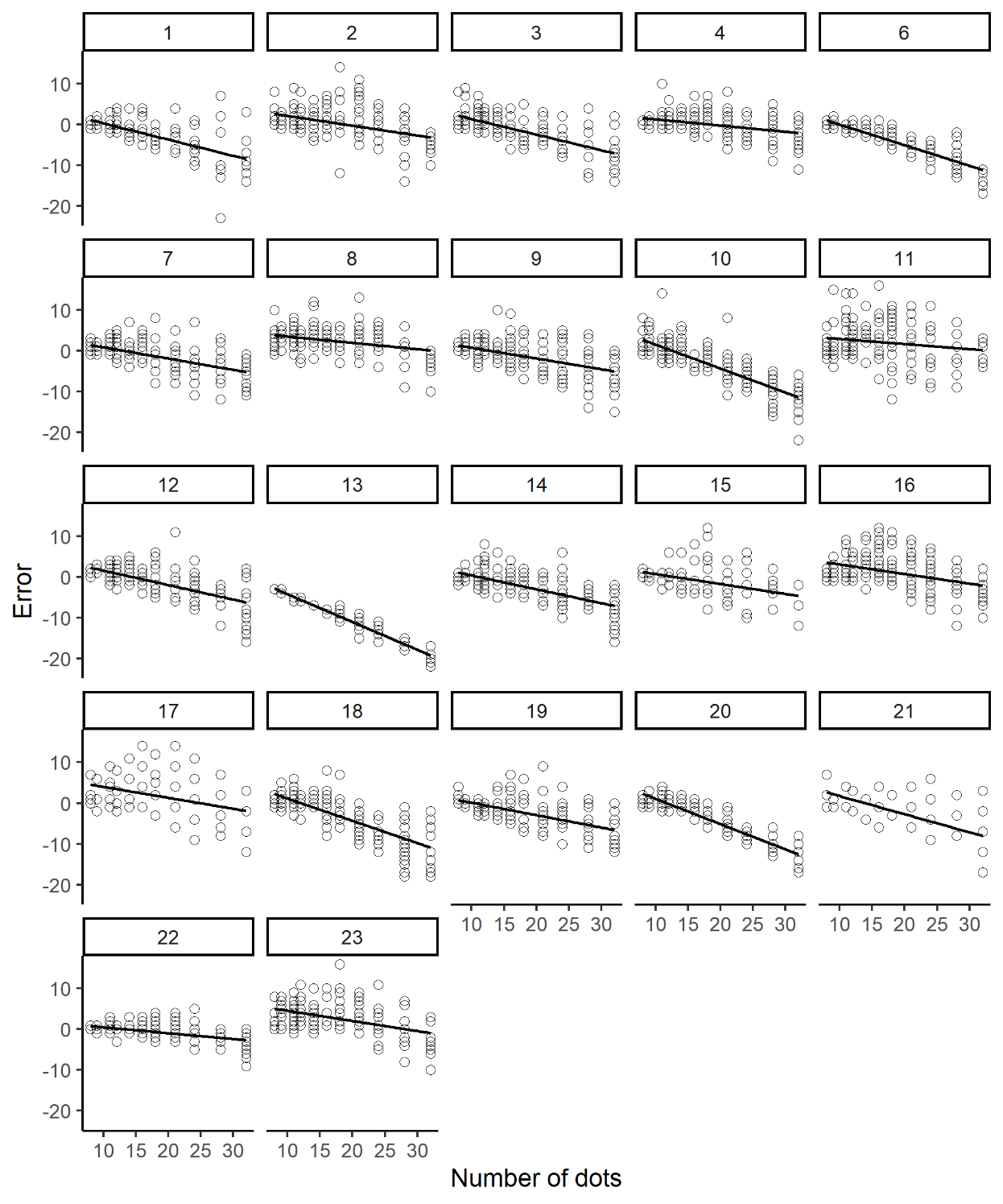


# **Figure S5.** Distribution of errors as a function of the number of dots in the current trial immediately after the response trial in Experiment 2. The circles in the graph indicate the error value for each participant on each trial. The numbers in the rectangles represent the participant number. The lines in the graphs indicate the slopes obtained from regression analyses.

#
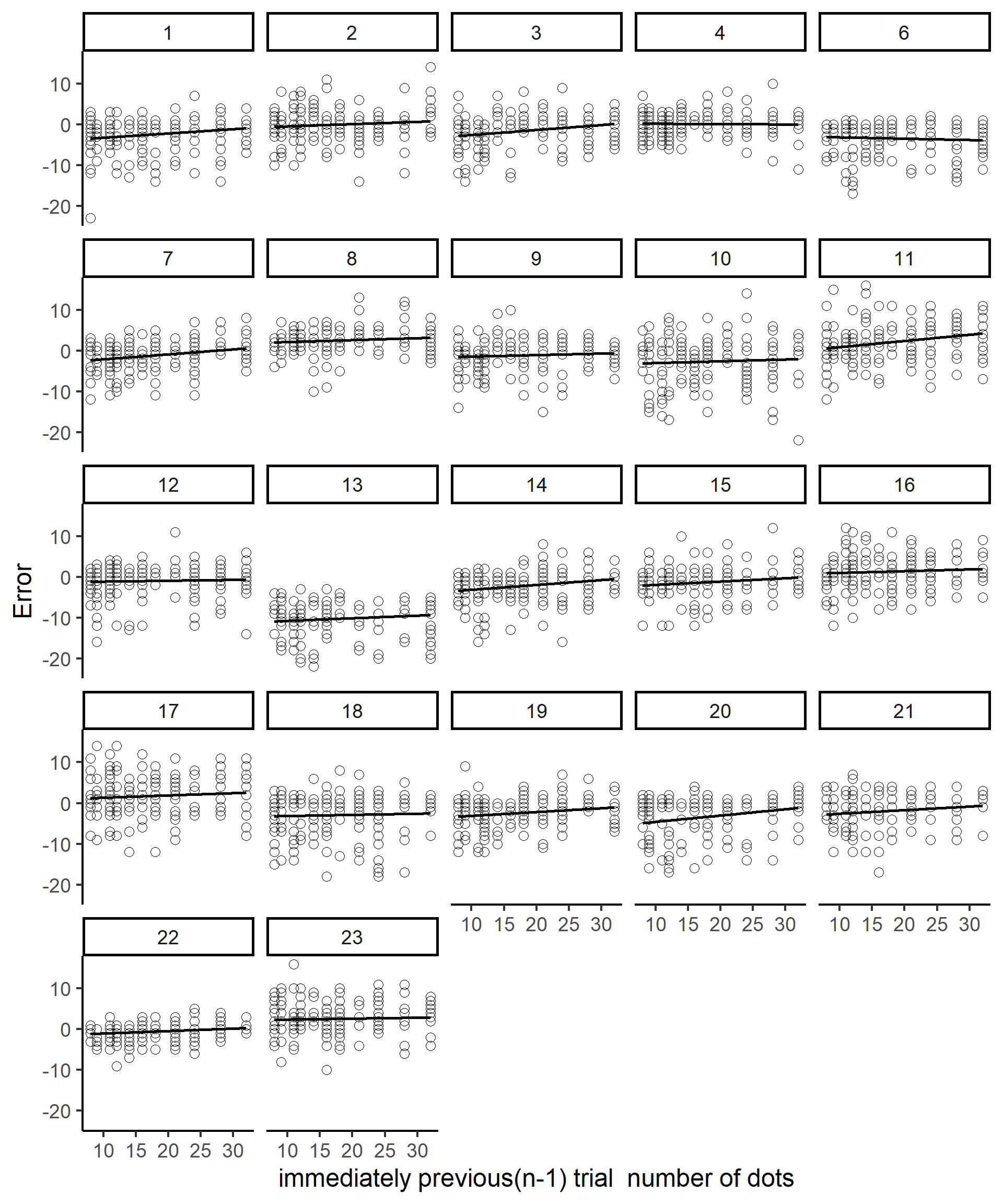


# **Figure S6.** Distribution of errors as a function of the number of dots in the n-1 trial immediately after the response trial in Experiment 2. The circles in the graph indicate the error value for each participant on each trial. The numbers in the rectangles represent the participant number. The lines in the graphs indicate the slopes obtained from regression analyses.


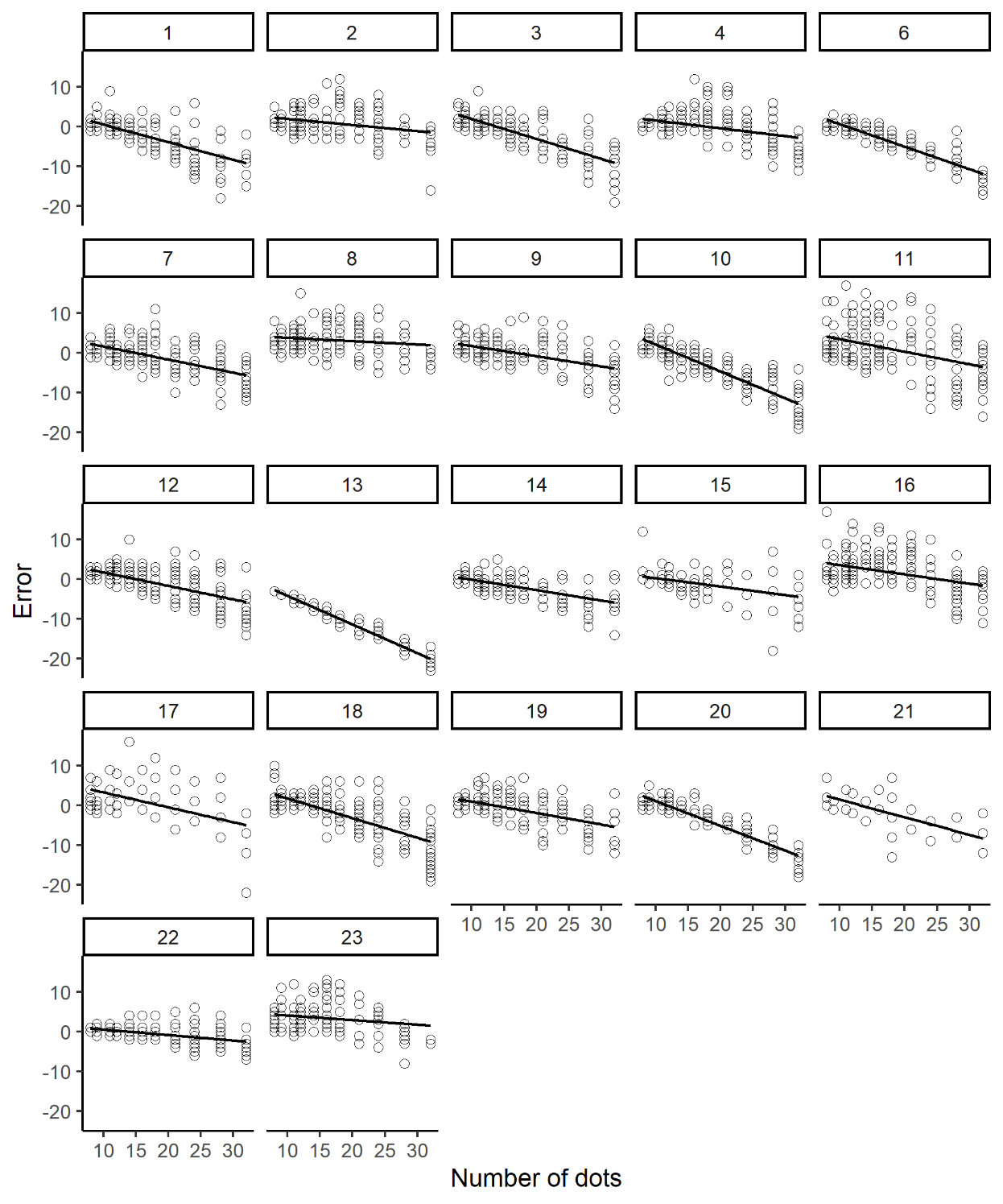


# **Figure S7.** Distribution of errors as a function of the number of dots in the current trial immediately after the no-response trial in Experiment 2. The circles in the graph indicate the error value for each participant on each trial. The numbers in the rectangles represent the participant number. The lines in the graphs indicate the slopes obtained from regression analyses.


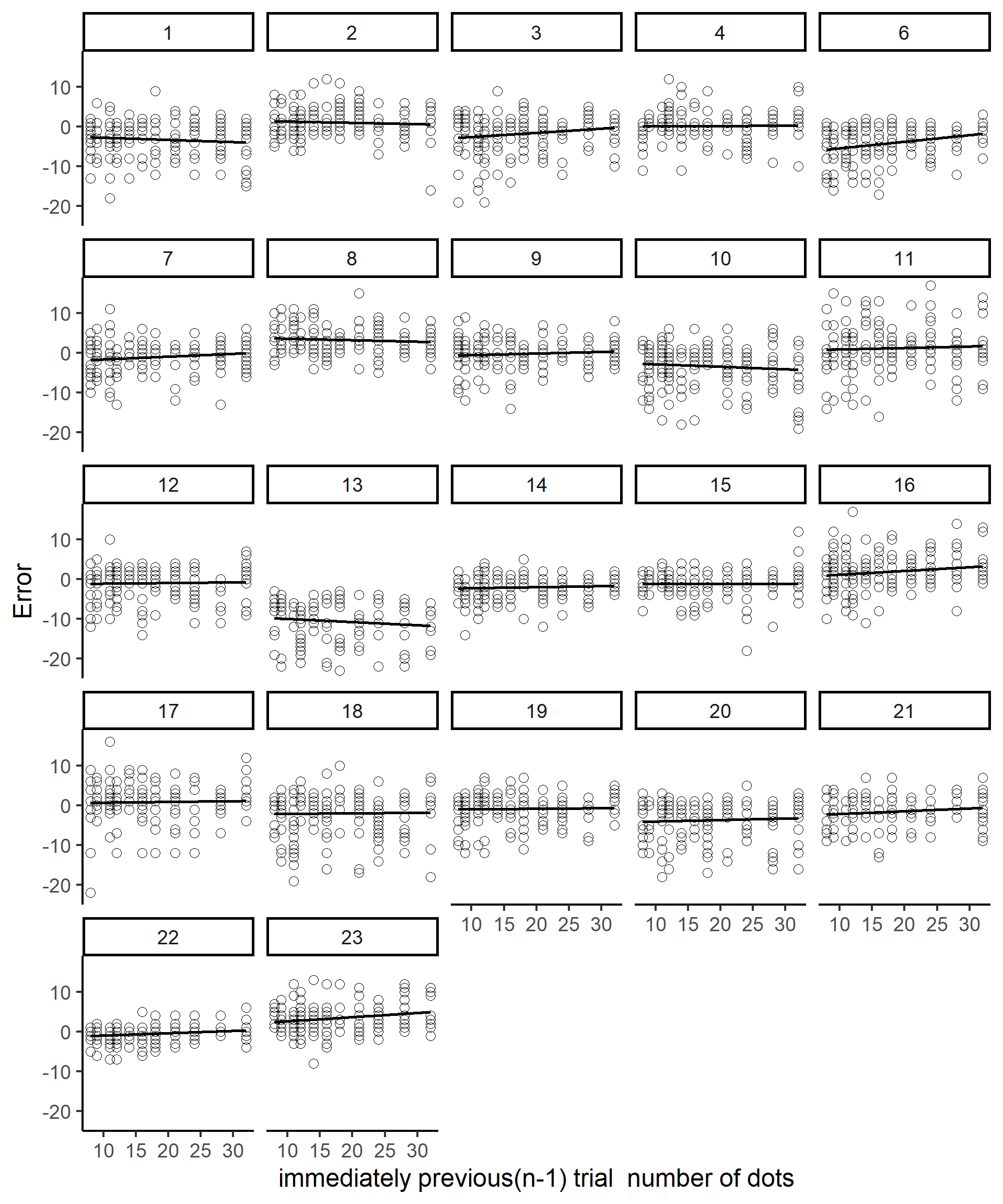


# **Figure S8.** Distribution of errors as a function of the number of dots in the n-1 trial immediately after the no-response trial in Experiment 2. The circles in the graph indicate the error value for each participant on each trial. The numbers in the rectangles represent the participant number. The lines in the graphs indicate the slopes obtained from regression analyses.

# As an additional analysis, we performed regression analyses using the number of dots or the participant's response from n-3 to n+1 trials as explanatory variables (factors in the table). Because the number of tests differs from the analyses in the main text, the *Adj.p* values are different from those in the main text.

## Experiment 1a

**Table S1.** Summary of serial dependence effect (single regression analyses) for Experiment 1a.

NoD indicates the number of dos in the stimuli and Ans indicates the participant’s response.

| **Factor** | **Average effect** | **95% CI** | **N** | ***t* value** | ***d*** | ***Adj.p* value** |  | **Mean**  **adj.R^2^** |
| --- | --- | --- | --- | --- | --- | --- | --- | --- |
| n-1 NoD | 0.058 | 0.014 | 32 | 8.65 | 1.51 | 0.001 < | *** | 0.01 |
| n-2 NoD | 0.013 | 0.013 | 32 | 2.08 | 0.36 | 0.057 | n.s. | 0.00 |
| n-3 NoD | 0.002 | 0.010 | 32 | 0.32 | 0.06 | 0.837 | n.s. | 0.00 |
| n NoD | -0.358 | 0.055 | 32 | -13.24 | -2.31 | 0.001 < | *** | 0.44 |
| n+1 NoD | 0.001 | 0.010 | 32 | 0.16 | 0.03 | 0.877 | n.s. | 0.00 |
| n-1 Ans | 0.123 | 0.021 | 32 | 11.65 | 2.03 | 0.001 < | *** | 0.03 |
| n-2 Ans | 0.046 | 0.020 | 32 | 4.63 | 0.81 | 0.001 < | *** | 0.01 |
| n-3 Ans | 0.028 | 0.017 | 32 | 3.31 | 0.58 | 0.004 | ** | 0.00 |
| Ans | -0.150 | 0.105 | 32 | -2.90 | -0.51 | 0.010 | * | 0.11 |
| n+1Ans | 0.028 | 0.015 | 32 | 3.76 | 0.66 | 0.001 | ** | 0.00 |

## Experiment 1b

**Table S2.** Summary of serial dependence effect (single regression analyses) for Experiment 1b.

NoD indicates the number of dos and Ans indicates the participant’s response.

| **Factor** | **Average effect** | **95% CI** | **N** | ***t* value** | ***d*** | ***Adj.p* value** |  | **Mean**  **adj.R^2^** |
| --- | --- | --- | --- | --- | --- | --- | --- | --- |
| n-1 NoD | 0.001 | 0.014 | 32 | 0.090 | 0.016 | 0.929 | n.s. | 0.002 |
| n-2 NoD | 0.029 | 0.016 | 32 | 3.663 | 0.638 | 0.003 | ** | 0.007 |
| n-3 NoD | -0.011 | 0.012 | 32 | -1.872 | -0.326 | 0.101 | n.s. | 0.001 |
| n NoD | -0.334 | 0.053 | 32 | -12.934 | -2.252 | 0.001 < | *** | 0.410 |
| n+1 NoD | -0.010 | 0.013 | 32 | -1.526 | -0.266 | 0.171 | n.s. | 0.002 |
| n-2 Ans | 0.072 | 0.021 | 32 | 6.949 | 1.210 | 0.001 < | *** | 0.013 |
| n-4 Ans | 0.030 | 0.017 | 32 | 3.474 | 0.605 | 0.004 | ** | 0.002 |
| n-6 Ans | 0.014 | 0.014 | 32 | 1.903 | 0.331 | 0.101 | n.s. | 0.000 |
| Ans | -0.139 | 0.103 | 32 | -2.736 | -0.476 | 0.020 | * | 0.101 |
| n+2Ans | 0.010 | 0.022 | 32 | 0.945 | 0.165 | 0.391 | n.s. | 0.003 |

## Experiment 2

**Table S3.** Summary of serial dependence effect (single regression analyses) for Experiment 2 immediately after the response trial. NoD indicates the number of dos and Ans indicates the participant’s response.

| **Factor** | **Average effect** | **95% CI** | **N** | ***t* value** | ***d*** | ***Adj.p* value** |  | **Mean**  **adj.R^2^** |
| --- | --- | --- | --- | --- | --- | --- | --- | --- |
| n-1 NoD | 0.067 | 0.022 | 21 | 6.44 | 1.37 | 0.001 < | *** | 0.017 |
| n_NoD | -0.342 | 0.072 | 21 | -9.89 | -2.11 | 0.001 < | *** | 0.407 |
| n-1 Ans | 0.125 | 0.041 | 21 | 6.31 | 1.34 | 0.001 < | *** | 0.032 |
| n Ans | -0.176 | 0.187 | 21 | -1.95 | -0.42 | 0.064 | n.s. | 0.103 |
